# Multimodal classification of extremely preterm and term adolescents using the fusiform gyrus: A machine learning approach

**DOI:** 10.1016/j.nicl.2022.103078

**Published:** 2022-06-04

**Authors:** Connor Grannis, Andy Hung, Roberto C. French, Whitney I. Mattson, Xiaoxue Fu, Kristen R. Hoskinson, H. Gerry Taylor, Eric E. Nelson

**Affiliations:** a Center for Biobehavioral Health, Abigail Wexner Research Institute, Nationwide Children’s Hospital, Columbus, OH, United States; b Department of Pediatrics, Ohio State University Wexner College of Medicine, Columbus, OH, United States; c College of Education, University of South Carolina, Columbia, SC, United States

**Keywords:** Adolescence, Preterm birth, Face processing, Brain connectivity

## Abstract

**Objective:**

Extremely preterm birth has been associated with atypical visual and neural processing of faces, as well as differences in gray matter structure in visual processing areas relative to full-term peers. In particular, the right fusiform gyrus, a core visual area involved in face processing, has been shown to have structural and functional differences between preterm and full-term individuals from childhood through early adulthood. The current study used multiple neuroimaging modalities to build a machine learning model based on the right fusiform gyrus to classify extremely preterm birth status.

**Method:**

Extremely preterm adolescents (n = 20) and full-term peers (n = 24) underwent structural and functional magnetic resonance imaging. Group differences in gray matter density, measured via voxel-based morphometry (VBM), and blood-oxygen level-dependent (BOLD) response to face stimuli were explored within the right fusiform. Using group difference clusters as seed regions, analyses investigating outgoing white matter streamlines, regional homogeneity, and functional connectivity during a face processing task and at rest were conducted. A data driven approach was utilized to determine the most discriminative combination of these features within a linear support vector machine classifier.

**Results:**

Group differences in two partially overlapping clusters emerged: one from the VBM analysis showing less density in the extremely preterm cohort and one from BOLD response to faces showing greater activation in the extremely preterm relative to full-term youth. A classifier fit to the data from the cluster identified in the BOLD analysis achieved an accuracy score of 88.64% when BOLD, gray matter density, regional homogeneity, and functional connectivity during the task and at rest were included. A classifier fit to the data from the cluster identified in the VBM analysis achieved an accuracy score of 95.45% when only BOLD, gray matter density, and regional homogeneity were included.

**Conclusion:**

Consistent with previous findings, we observed neural differences in extremely preterm youth in an area that plays an important role in face processing. Multimodal analyses revealed differences in structure, function, and connectivity that, when taken together, accurately distinguish extremely preterm from full-term born youth. Our findings suggest a compensatory role of the fusiform where less dense gray matter is countered by increased local BOLD signal. Importantly, sub-threshold differences in many modalities within the same region were informative when distinguishing between extremely preterm and full-term youth.

## Highlights

• Structural and functional differences found in right fusiform of preterm youth.
• Both reduced gray matter and enhanced BOLD signal to faces was found.
• A classifier using density, tractography and connectivity surpassed 95% accuracy.
• Increased BOLD may be compensatory response for gray matter reductions.

## 1 Introduction

Preterm birth, defined as birth at a gestational age < 37 weeks, is a common occurrence, affecting roughly 10% of newborns in the United States (Rogers and Hintz, 2016). Although survival rates of preterm (PT) infants have improved substantially, both physical and behavioral morbidities are common in this population across development (Janvier et al., 2013). In late childhood and adolescence, peer integration is one domain in which behavioral difficulties are particularly evident (Taylor, 2020). Social difficulties, along with emotion regulation and attention problems, occur consistently in preterm populations and comprise the three prongs of the ‘preterm behavioral phenotype’ that characterizes developmental difficulties in preterm children (Johnson and Marlow, 2011). Specific dysfunction in social cognition and socio-emotional behavior are frequently reported even after other cognitive and emotional difficulties are accounted for (for review, see (Dean et al., 2021).

Over the past two decades, the neural substrates that underlie specific components of social cognition have been the focus of a great deal of neuroscience research (Stanley and Adolphs, 2013). Due to the extreme importance of sociality for the survival of social primates (including humans), specific neural systems are thought to have evolved for the processing of social signals—such as faces and voices—in addition to more complex social cognitive capacities like mentalizing, social valuation, and social memory (Adolphs, 2009, Blakemore, 2008, Brothers et al., 2002, Dunbar, 2009, Rushworth et al., 2013). Indeed, in addition to the classic ventral (what) and dorsal (where) visual streams, a third pathway was recently proposed along the superior temporal lobe in both human and non-human primates. In this pathway, social percepts such as faces, body movements, and voices are thought to undergo neuronal processing which includes multimodal integration, valuation, and abstraction of social information (Pitcher and Ungerleider, 2021).

The fusiform face area (FFA) is one of the earliest pathways into this social information processing stream (Pitcher and Ungerleider, 2021). The FFA is a functionally defined region in the fusiform gyrus along the ventral temporal lobe that selectively responds to faces relative to other similar visual categories (Haxby et al., 2002, Kanwisher and Yovel, 2006). Substantial maturational refinements of the FFA occur across development and into adulthood (Germine et al., 2011, Nordt et al., 2018). However, exposure to faces in early infancy is thought to be a particularly important window for tuning FFA responsiveness to faces (Fox et al., 2011, Maurer and Werker, 2013, Sugita, 2008), rendering it potentially especially vulnerable to the impact of preterm birth.

Several recent studies have reported behavioral and perceptual differences in infants, children, and adults who were born preterm that may relate to altered fusiform function (Fenoglio et al., 2017, Hasler et al., 2020, Healy et al., 2013, Mathewson et al., 2020). Throughout development, individuals born PT demonstrate differential processing of faces and social scenes. PT infants and children exhibit both a lack of preference to social information relative to non-social information and a different social orienting profile, with PT infants looking at the mouth more often than the eyes relative to full-term (FT) infants (Telford et al., 2016). Specific deficits in face memory have also been found in PT infants (Perez-Roche et al., 2017). This profile persists through childhood and into adulthood, where individuals born PT tend to process visual stimuli by focusing on features in isolation (local configuration) rather than tending to relate individual features to the rest of the scene (holistic approach) (Mathewson et al., 2020, Pavlova et al., 2021, Santos et al., 2010). Local configural processing is associated with poorer performance IQ, which may suggest a deficit in perceptual processing (Mathewson et al., 2020).

In addition to these behavioral differences in face responding, several studies have also reported alterations in brain regions that underlie face processing, including structural differences in the fusiform gyri (Bäuml et al., 2015, Healy et al., 2013, Meng et al., 2016, Nosarti et al., 2008, Shang et al., 2019) , as well as other emotional face processing regions (Healy et al., 2013, Kesler et al., 2008, Nosarti et al., 2008, Peterson et al., 2000). While the direction of these differences are inconsistent, the right fusiform is continually mentioned as having differential gray matter volume than that of FT peers (Bäuml et al., 2015, Meng et al., 2016, Shang et al., 2019). Reduced functional responses have also been reported during exposure to faces in both PT infants (Frie et al., 2016) and older PT children (Mossad et al., 2020). In addition, alterations in the pattern of functional connectivity in social cognition regions has been demonstrated in PT individuals both at rest (Johns et al., 2019, Mossad et al., 2021, Papini et al., 2016) and during face processing tasks (Mossad et al., 2020, Sato et al., 2021) .

While these studies have separately demonstrated differences in structure or function of the fusiform gyrus, a few recent studies have also begun to integrate differences in both structure and function using machine learning approaches. These multivariate models can integrate information from multiple voxels and even modalities, resulting in a more holistic representation of how the included features are interrelated. Studies have found partly overlapping regions of altered structure and neural activity at rest in PT youth in both the right fusiform (Shang et al., 2019) and the right ventral attention network (Bäuml et al., 2015). However, these studies did not examine face-processing functions per se. To address this limitation, the current study examined both gray matter density and blood-oxygen level-dependent (BOLD) response during face-processing within the right fusiform. As an exploratory follow up, a linear support vector machine was employed to distinguish extremely preterm (EPT, gestational age < 28 weeks) from FT youth based on several metrics of the structure and function of the fusiform gyrus. These metrics included gray matter density, BOLD signal during a face processing task, the number of outgoing white matter streamlines from the right fusiform gyrus (rFG), and both local and brain wide functional connectivity during the task and at rest using the rFG as a seed region.

## 2 Material and methods

### 2.1 Participants

Fifty-four youth were recruited to participate in this study. Participants included EPT (gestational age < 28 weeks) and FT (gestational age >= 37 weeks) adolescents. EPT youth were identified through a review of electronic medical records at a large urban children’s hospital for youth with a diagnosis of preterm birth. EPT youth were mailed a letter of invitation and were given 10 days to opt out before being called to assess interest. FT youth were recruited via digital flyers distributed to hospital staff. Study procedures were approved by the local institutional review board and written informed consent/assent was obtained from all participants prior to participation. All procedures were evaluated and approved based on guidance from the Office of Human Research Protections, consistent with ethical principles and codes.

All participants were able to participate in the functional magnetic resonance imaging (fMRI) protocol, had normal or corrected vision, and were between the ages of 11–16 years (born between 2002 and 2008). Of the original 54 participants, 5 were excluded due to having a sibling also enrolled, four were excluded because they failed to complete the entire imaging protocol, and one was excluded due to a neurodevelopmental disorder. Of the participants with missing data, three were missing only resting state data, and one was missing data from all functional tasks. Importantly, although some radiologic abnormalities were detected in six participants (three in the EPT group), none had clinical MRI abnormalities in the rFG and all were kept in the analysis. Neurologic abnormalities were reported by a neuroradiologist and included pineal cyst, Chiari I, ventriculomegaly, and one case of encephalomalacia in the thalamic regions. The final sample consisted of 44 youth: 20 EPT and 24 FT youth. Basic demographic information of both groups including age, gender, race, ethnicity, birth weight, gestational age, and estimated yearly family income, a proxy for socioeconomic status, are reported in Table 1. Groups did not differ in age t(43) = 0.02, p = 0.99 or gender, χ2(1, N = 44) = 0.001, p = 0.97. All demographic variables were tested for homogeneity and all assumptions of normality were met except for birth weight (see Table 1).

**Table 1:** Displays demographic information for the extremely preterm and full-term groups. Age, gender, birth weight, gestational age, and family income are reported as mean (standard deviation); race and ethnicity data are reported as n (%). The “Non-White” racial category includes Black, Asian, and multi-race members. Birth weight and gestational age are both missing for 2 full-term participants. Birth weight is reported in grams. Gestational age is reported in weeks.

Demographic information				
	Preterm *M*(SD)	Full-term *M*(SD)	*t *scores	Levene’s Statistic
Age	13.45 (1.90)	13.46 (1.53)	0.02	0.19
Birth weight	807.96 (209.24)	3382.62 (348.02)	28.69**	4.67*
Gestational age	25.86 (1.26)	39.26 (1.12)	36.71**	0.49
Median family income	90578.83 (34412.59)	71312.00 (32293.57)	1.90	0.27

**Gender, racial, and ethnic information**				

	**Preterm n (%)**	**Full-term n (%)**	**χ2**	

Gender			0.08	
Female	10 (50.00)	13 (54.17)		
Male	10 (50.00)	11 (45.83)		
Race			6.19*	
Non-White	15 (75.00)	9 (37.50)		
White	5 (25.00)	15 (62.50)		
Ethnicity			1.55	
Hispanic/Latinx	3 (15.00)	1 (4.17)		
Non-Hispanic/Latinx	17 (85.00)	23 (95.83)		

### 2.2 MRI data acquisition

MRI data were collected on a Siemens 3 Tesla Prisma scanner using a 64-channel head coil. The imaging protocol included a whole brain isotropic 3D T1-weighted anatomical scan (Magnetization prepared-rapid gradient echo; MPRAGE), a single shell acquisition diffusion weighted imaging (DWI) for tractographic analysis, and fMRI using echo planar imaging (EPI) acquisitions. All sequences included simultaneous multi-slice acquisition. Imaging parameters for MPRAGE were: 1 mm^3^ voxels, 160 sagittal slices, repetition time (TR) = 2300 ms, echo time (TE) = 2.98 ms, field of view (FOV) = 240 mm^2^. DWI sequences were collected in 64 directions with b = 0, TR = 1900 ms, TE = 62 ms, FOV = 240 mm^2^, 36 axial slices, and 0.92 mm × 0.92 mm × 4 mm voxels. Parameters for the functional scans were: 2.5 mm × 2.5 mm × 4 mm voxels, 36 axial slices, TR = 1500 ms, TE = 30 ms, FOV = 240 mm^2^. The face task (see section 2.3) involved 532 brain volumes acquired across two 6.5-minute runs. 246 brain volumes of resting state were also acquired using the same parameters in a separate single 6.25-minute run. Due to changes made part way through data collection, a subset of 5 full-term participants’ resting state images were acquired using slightly different parameters: 3 mm × 3 mm × 4 mm voxels, 48 axial slices, TR = 2000 ms, TE = 28 ms, FOV = 240 mm^2^. 160 brain volumes were collected in a single 5.33-minute run. For the rest sequence, a visual fixation cross was displayed, and participants were instructed to keep their eyes open and rest for the entire sequence.

### 2.3 Neuroimaging task

The face processing task was an adaptation of a neuroimaging protocol used previously to assess neural systems employed during implicit judgements of trustworthiness and dominance (Engell et al., 2007, Oosterhof and Todorov, 2008, Todorov et al., 2011)). Stimuli were computer generated faces that varied on dimensions of dominance and trustworthiness from a large publicly available sample (Oosterhof and Todorov, 2008); https://tlab.uchicago.edu/). The task was ostensibly a face recognition task in which a set of 10 stimulus faces were shown followed by a single test face. Participants were asked to indicate via button press if the test face had appeared in the previous set of probes. All faces were presented for a 1 s duration. The inter-trial interval between stimulus faces was randomly set to 1.5 or 3.5 s and a 3 s response interval followed the test face. In total, 140 stimulus and 14 test faces were administered (see S1 for diagram). Both test and stimulus faces varied on dominance and trustworthiness dimensions. For the present purposes, all faces were collapsed across these dimensions and treated as a single category.

### 2.4 Data processing and analysis

#### 2.4.1 T1-and DWI images

The T1-weighted MPRAGE images underwent standard processing using the current version of Statistical Parametric Mapping (SPM12) software (Ashburner and Friston, 2000). This included 1) manual re-alignment to the anterior commissure, 2) segmentation into gray matter, white matter, and cerebrospinal fluid, 3) image registration, normalization, and modulation (Ashburner, 2007), 4) transformation to Montreal Neurological Institute (MNI) space, and 5) smoothing with a 10 mm full width at half maximum (FWHM) isotropic Gaussian kernel. Group differences in gray matter density, measured via voxel-based morphometry (VBM), were then compared with a two-sample *t-*test that was masked to only include the rFG from the automated anatomical labeling (AAL) atlas (Tzourio-Mazoyer et al., 2002). Estimated total intracranial volume (ETIV) was calculated by adding gray matter, white matter, and cerebrospinal fluid volume for each participant. ETIV was included as a regressor of non-interest in the SPM12 second-level analyses. The results from these analyses generated a density map. Results were considered significant if they passed family-wise error correction at *p* < 0.05.

DWI images underwent standard preprocessing including correction for both eddy current and motion with FSL’s fMRIB Diffusion Toolbox (v5.0). This was followed by probabilistic tractocographic analysis using Bayesian Estimation of Diffusion Parameters Obtained using Sampling Techniques (BEDPOSTx), which established diffusion parameters at each voxel (Behrens et al., 2007). A single b0 was obtained with the remaining 128b-values equal to 1000.

#### 2.4.2 Functional imaging

EPI images from both the task and resting state scans were denoised using Automatic Removal of Motion Artifacts (AROMA) as described in (Pruim et al., 2015) and further preprocessed using standard pipelines in Analysis of Functional NeuroImaging (AFNI), version 18.1.02 and analyzed in AFNI, version 20.3.01 (Cox, 1996). Images were aligned to the anterior commissure/posterior commissure plane, co-registered to the T1 image, normalized non-linearly to the MNI template, and spatially smoothed with a Gaussian filter (FWHM, 6 mm kernel). Motion outliers were identified as volumes exceeding 1 mm Euclidean distance based on framewise change. Volumes in which more than 10% of voxels were signal outliers (as defined by AFNI 3dToutcount, based on median absolute deviation from the time series) were regressed out of the final model. Voxel-wise signal was scaled to a mean value of 100 and signal values above 200 were winsorized to 200 within each run. Nuisance regressors for motion (6 affine directions and their first-order derivatives) and scanner drift (third polynomial) were also included.

For task-based analysis, the hemodynamic response function was convolved with a basis function for the duration of face presentation. A separate regressor for test faces was included but test faces were regressed out of analyses. The fixation cross was displayed during inter-trial intervals and served as the implicit baseline: contrasts were thus non-test face stimuli relative to baseline. Although the stimuli in this task were designed to probe implicit responses to the dominance and trustworthiness of faces, the present analysis was focused on more rudimentary face processing, so we collapsed across dominance and trustworthiness dimensions and included only a single regressor for face presentation, excluding test faces, to maximize power. AFNI’s 3dttest++ was used to generate group comparisons using the face minus baseline contrast images produced at the individual participant level. Cluster size threshold corrections were estimated using the AFNI command 3dClustsim, with two-sided thresholding and first-nearest neighbor clustering at α = 0.05 and *p* < 0.005. The resulting cluster threshold of 33 voxels was applied to the results.

#### 2.4.3 Secondary analyses

Primary analysis involved assessment of group differences in gray matter density and BOLD response to faces in the rFG. Because significant group differences were found on both measures, we further probed group differences with a series of secondary analyses using white matter tractography, regional homogeneity, and functional connectivity of BOLD signals during both task and rest. Results from all analyses were then included in machine learning classification analyses. These analyses were conducted to investigate how these features are interrelated with each other, yielding a more holistic depiction of differences in the rFG.

##### 2.4.3.1 Functional connectivity during task

Functional connectivity during the face processing task was examined using generalized psychophysiological interaction (gPPI) analyses (McLaren et al., 2012). First, we used the observed significant group difference clusters in density and BOLD responses to define gPPI seed regions within the rFG. We then fit the same subject-level model (faces vs baseline) with the addition of activation in this seed, and an interaction between the seed and task to identify regions that were co-activated alongside the seed region. Whole brain group-level comparisons using AFNI’s 3dttest++ were then performed comparing these co-active regions.

The resulting *t*-maps were masked to exclude the seed region and an uncorrected cluster-forming threshold of *p* < 0.005 and cluster size of 50 voxels was used to reduce spurious findings.

##### 2.4.3.2 Functional connectivity at rest

A seed-based approach was used to analyze resting state functional connectivity (RS-FC) using AFNI’s 3dGroupIncorr. As in the gPPI approach above, group difference clusters identified in the BOLD and VBM analyses were used as seed regions and the mean time series within each seed was correlated with the time series of all other voxels in the brain. Group difference clusters were formed via two-sample *t*-tests of FT minus EPT. To reduce spurious findings, a cluster-forming threshold of *p* < 0.005 and cluster size of 50 voxels was used. Brain-wide group differences in both task-based and resting state connectivity patterns were subsequently input into the classifier model.

##### 2.4.3.3 White matter connectivity

An ROI-based tractography approach was used to calculate white matter connectivity from the group difference clusters from the BOLD and VBM analyses (seeds) to all other regions. Probabilistic tractography (probtrackx2; (Behrens et al., 2007)) was used to determine streamline counts leaving each seed and terminating in any cortex gray matter (as labeled by Desikan-Killiany atlas (Desikan et al., 2006) after applying an exclusion mask of the right fusiform AAL atlas region to avoid short range white matter connections. In this calculation, we enabled distance correction; in other words, the distance between each streamline’s origin and terminating location was used to weight the streamline count to avoid bias toward lower counts on longer-distance connections (Behrens et al., 2007).

##### 2.4.3.4 Regional homogeneity

Functional connectivity at rest among neighboring voxels was calculated using AFNI’s 3dReHo (Taylor and Saad, 2013) function. Regional homogeneity (ReHo) is a measure of similarity among neighboring voxels during fMRI. For each voxel within the seed region, ReHo was defined as the Kendall’s coefficient of concordance (KCC) of the time series including first-nearest neighbors, with higher values indicating higher temporal synchronization within those voxels and suggesting functional integrity of clusters. At the subject level, ReHo was calculated for each voxel within the seed regions, then smoothed with a Gaussian filter (FWHM, 6 mm kernel). Altered ReHo has been reported in several clinical populations, suggesting disrupted communication among neighboring voxels (Cao et al., 2006, Liu et al., 2006, Liu et al., 2008). Additionally, there is evidence that many clusters of altered ReHo are positively correlated with alterations in gray matter density (Wang et al., 2012), revealing an important relationship that is well suited for multivariate analyses to further probe; in other words, more dense gray matter has been related to stronger temporal synchronization and including both of these features in a classification model may yield better results than if only one feature (density or ReHo) is included.

#### 2.4.4 Support vector machine classifications

Finally, group difference clusters from the primary VBM and BOLD analyses were further probed using a linear support vector machine classifier (SVC). Data from all six modalities (gray matter density, BOLD signal, ReHo, outgoing white matter streamlines, and functional connectivity during the task and at rest) were recomputed within each cluster and were included to investigate the impact of multiple modalities from the rFG on the classification of EPT and FT youth. Group difference clusters from the primary analyses were probed in isolation to examine local differences within the rFG. Classifiers were built in Python 3.8 using the default parameters in Scikit-Learn (version 0.23.1) (Pedregosa et al., 2011). For each cluster identified in the primary analyses, input into the classifier included the recomputed 1) average BOLD signal to all faces; 2) average gray matter density; 3) average ReHo; 4) number of outgoing white matter streamlines; 5) functional connectivity between the cluster and any regions surviving the gPPI analysis; and 6) RS-FC between the cluster and any regions surviving the resting state analysis.

All modalities were included in the classifications, regardless of significant univariate differences: thresholding was employed for the gPPI and RS-FC analyses solely as a feature reduction technique. Since gray matter density, BOLD, tractography, and ReHo analyses yielded one result per cluster instead of a whole brain *t*-map, no thresholding was necessary. By building multimodal classifiers and observing how adding data modalities can affect classification performance, we can understand whether different variables carry redundant or complementary information and identify those features that better differentiate the extremely preterm phenotype. A total of 32 classifiers, each representing different feature combinations, were evaluated for each cluster (see S2 and S3 for a full breakdown of classifier performance). These feature combinations consisted of the orthogonal combinations of all modalities as long as the primary cluster-derived modality was also included. Despite having binary target labels, the “chance” level in these complex data structures may not be equivalent to 50 percent: in such cases, permutation testing can be employed to address this issue. Permutation testing is a non-parametric bootstrapping technique where the target labels (EPT or FT) are randomly shuffled to generate a null distribution. Our true accuracy was tested against this null distribution created from 1000 random iterations to obtain a *p*-value.

The resulting classifier input was a *z*-scored 44-subject by 6-feature matrix. Model performance was evaluated using a leave-one-participant-out cross-validation scheme. For each cluster, all combinations of features that included the modality from the primary cluster-forming analysis (i.e., VBM or BOLD) were evaluated to find which combination of features was best at distinguishing EPT from FT youth.

## 3 Results

### 3.1 Primary analyses

#### 3.1.1 Behavioral task performance

Although behavioral performance was not considered in any analyses, group differences were compared to confirm similar levels of engagement between groups. Independent *t*-tests revealed no group differences in either behavioral accuracy *t*(43) = 1.04, *p* = 0.31 or reaction time *t*(43) = -0.03, *p* = 0. 98 across task. Levene’s test indicated equal variances between groups for both behavioral accuracy (*p* = 0.69) and reaction time (*p* = 0.71).

#### 3.1.2 Local neural response to face perception

**Fig. 1:**
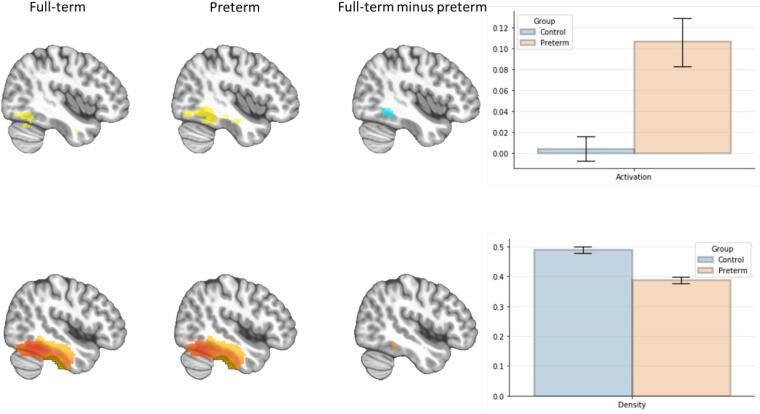
The mean differences in BOLD signal during a face processing task (top) and grey matter density (bottom) in the right fusiform. Full-term youth show a more restricted area of activation while the extremely preterm youth have stronger, more widespread activation. In contrast to the activation patterns, full-term youth had a cluster of more dense grey matter. Warmer colors in the brain images indicate areas in which full-term youth had relatively higher values compared to extremely preterm youth.

Comparison of BOLD response to faces vs baseline within the rFG revealed a cluster of *greater* activation in the EPT compared to FT youth (see Table 2 and Fig. 1).

**Table 2:** Displays the peak coordinates and *t*-scores for the BOLD (a) and VBM (b) analyses. Both analyses are set up as full-term minus extremely preterm. (c) displays the peak coordinates and *t*-score for the prefrontal cortex (PFC) cluster resulting from the gPPI analysis using the seed region resulting from the VBM analysis.

(a) R Fusiform BOLD response to neutral faces						
Region	X	Y	Z	Cluster size	T value	P value
R Fusiform	−45	47	−15	67	−3.50	0.01

**(b) R Fusiform gray matter density**						

**Region**	**X**	**Y**	**Z**	**Cluster size**	**T value**	**P value**

R Fusiform	−46	−42	−18	38	5.56	0.01

**(c) PFC cluster from gPPI analysis**						

**Region**	**X**	**Y**	**Z**	**Cluster size**	**T value**	**P value**

PFC	–23	−68	16	130	3.57	< 0.005*

* The p-value reported for the PFC region from the gPPI analysis is uncorrected and does not survive cluster correction at α = 0.05.

#### 3.1.3 Gray matter density

A cluster within the rFG that partially overlapped with the cluster of BOLD differences revealed the opposite pattern: greater density in FT relative to EPT youth (see Table 2 and Fig. 1).

### 3.2 Secondary analyses

#### 3.2.1 Functional connectivity during task and at rest

Functional connectivity during the face processing task and at rest were evaluated for each cluster found in the primary analyses. No regions survived thresholding for the RS-FC analysis using either cluster as the seed region. Similarly, no regions survived thresholding in the gPPI analysis for the cluster identified from the BOLD analysis. However, a region in the prefrontal cortex (PFC) (see Table 2) survived the gPPI analysis when the seed from the VBM analyses was used. For consistency in the subsequent classifiers, results from each seed were masked using this identified PFC region from the gPPI analysis.

For both clusters from the primary analyses, there were non-significant trends of greater RS-FC in FT relative to EPT youth. Similarly, functional connectivity during the face processing task was non-significantly greater in FT relative to EPT youth in the cluster identified in the primary VBM analysis.

#### 3.2.2 White matter connectivity

The number of outgoing white matter streamlines were calculated per person for each cluster from the primary analyses. Independent *t*-tests revealed no significant differences between groups in the cluster from the face processing task *t*(43) = 1.15, *p* = 0.26 or the cluster from the VBM task *t*(43) = 0.56, *p* = 0.58. Levene’s statistic indicated equal variances between groups for the number of outgoing white matter streamlines emanating from the cluster from the face processing task (*p* = 0.81) and the cluster from the VBM task (*p =* 0.52).

#### 3.2.3 Regional homogeneity

ReHo was used to measure the functional integrity among neighboring voxels during the resting state scan. Independent *t*-tests revealed no significant differences between groups in the cluster from the face processing task *t*(43) = 0.30, *p* = 0.77 or the cluster from the VBM task *t*(43) = −1.26, *p* = 0.21. Levene’s statistic indicated equal variances between groups for the functional integrity of the cluster from the face processing task (*p* = 0.98), however unequal variances were indicated in the cluster from the VBM task (*p =* 0.04).

#### 3.2.4 Classifier performance

##### 3.2.4.1 Classification in the cluster of BOLD signal differences

**Fig. 2:**
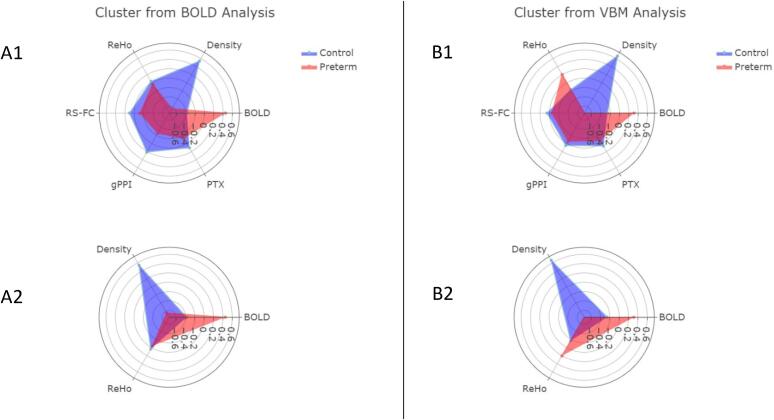
Radar plots represent the *z*-scored data for each modality. The left column (A) shows plots from the cluster of BOLD differences in the primary analysis. The right column (B) shows plots from the cluster of VBM differences in the primary analysis. The top row (1) displays the mean standardized value for each modality and the bottom row (2) displays the mean standardized value for only gray matter density, BOLD signal, and ReHo. A1 was the best combination of features for the cluster identified from the primary BOLD analysis, with a classification accuracy of 88.64%. B2 was the best combination of features for the cluster identified from the primary VBM analysis, with a classification accuracy of 95.45%.

When BOLD signal was considered in isolation for the cluster identified from the face processing task, the model had an accuracy score of 77.27% (SD = 41.90, *p* < 0.001). The addition of gray matter density increased accuracy to 79.55% (SD = 40.34, *p* < 0.001). The further inclusion of ReHo and outgoing white matter streamlines yielded an accuracy of 84.09% (SD = 36.58, *p* < 0.001), and peak performance was achieved when all six modalities were included in the model with an accuracy of 88.64% (SD = 31.74, *p* < 0.001) (see Fig. 2a). The best performing classifier was re-computed excluding the two participants for which birth status was missing but assumed to be full-term (accuracy = 85.71, SD = 36.46, *p*-value < 0.001). Separately, this classifier was re-computed excluding the six participants with neurological abnormalities (accuracy = 84.21%, SD = 36.46, *p*-value < 0.01).

##### 3.2.4.2 Classification in the cluster of gray matter differences

When gray matter density was considered in isolation for the cluster identified from the density analysis, the model had an accuracy of 88.64% (SD = 31.74, *p* < 0.001). The next highest accuracy score was achieved when ReHo was added to the model, giving an accuracy score of 93.18% (SD = 25.21, *p* < 0.001). Peak performance was attained when density, ReHo, and BOLD were included in the model with an accuracy score of 95.45% (SD = 20.83, *p* < 0.001). When all six modalities were included, the model was only 84.09% (SD = 36.58, *p* < 0.001) accurate suggesting suboptimal overfitting of parameters (see Fig. 2b). The best performing classifier was re-computed excluding the two participants for which birth status was missing but assumed to be full-term (accuracy = 95.24, SD = 21.30, *p*-value < 0.001). Separately, this classifier was re-computed excluding the six participants with neurological abnormalities (accuracy = 94.74, SD = 22.33, *p*-value < 0.001).

##### 3.2.4.3 Validation analyses

As a validation to our results, we recomputed the primary, secondary, and classification analyses within a leave-one-participant-out framework to control for data from the training set influencing the accuracy of the test set. Results from this validation are consistent with our reported results and a break-down of model performance is included in Supplemental Materials (S4). As an additional validation examining the specificity of the right fusiform, secondary and classification analyses were computed within the left supplementary motor area, which we did not expect to be as discriminatory as the rFG. Classification accuracy within this region was drastically lower than in the rFG (see S5).

## 4 Discussion

Building on previous studies demonstrating that preterm birth is associated with alterations in behavioral performance and brain responses to perceptual processing of faces, the current study assessed the ability of machine learning to classify individuals into EPT or FT groups based solely on information from the rFG, a region important for face processing. Consistent with previous studies, we found differences in both structural and functional responses in the rFG of EPT and FT adolescents using unimodal analyses. Results from the primary analyses revealed that EPT youth had *less* gray matter density and *greater* BOLD signal in response to faces compared to the FT youth. In addition, we were able to demonstrate a remarkable 95% classification accuracy using a variety of additional structural and functional metrics from this brain region.

Beyond the negative relationship between density and BOLD signal, the rFG showed different spatial patterns in BOLD response between groups. The neural response to faces was more localized in the FT youth and more widespread in the EPT youth (see Fig. 1). This pattern of activation paired with the finding of less dense gray matter and lack of task differences may suggest the rFG of EPT youth is more inefficient than that of FT youth. Although speculative, decreased gray matter density may necessitate the need for increased (more widespread) neural resources to generate similar levels of functional perception.

Our speculation of inefficiency of the fusiform in EPT youth is further supported by our multimodal analyses (see Fig. 2). Findings of more focalized BOLD signal, more gray matter density, stronger functional connectivity to the PFC, and slightly more outgoing white matter streamlines may reflect greater efficiency of the fusiform in our FT sample. While this interpretation is consistent with our findings, fiber tracts are sensitive to many factors and increased white matter connectivity might not necessarily represent greater efficiency and may also reflect a greater number of outgoing streamlines, which may be interpreted as decreased efficiency. An interesting observation is the increase in ReHo within the rFG in the EPT youth in the cluster identified from the primary VBM analysis. While this should be interpreted cautiously because it did not reach significance, the findings of greater BOLD signal and greater functional integrity (as measured by ReHo) in EPT youth may both reflect compensation for reductions in gray matter density. Additionally, the lack of connectivity to the PFC and relatively fewer outgoing white matter streamlines may support this interpretation of more intensive local processing and inefficient distribution to other brain areas. A similar pattern of structural reductions and compensatory BOLD hyperactivity has been reported in PT adults during a working memory task (Froudist-Walsh et al., 2015). Thus, this pattern may be a common form of ‘compensatory plasticity’ throughout the brain.

Compensatory plasticity enables PT youth to function reasonably well under most circumstances, as evidenced by our lack of behavioral differences during the face processing task. However, subtle group differences often appear when tasks become more challenging (Mathewson et al., 2020, Stern, 2002). For example, differences in social processing during difficult tasks, such as biological motion tasks, have been reported (Taylor et al., 2009). These basic perceptual skills may have downstream consequences on more complex and slowly developing aspects of social cognition, like empathy and theory of mind, which some studies have found to be challenging for many PT children (Dean et al., 2021, Mossad et al., 2017). Further, the fusiform gyrus is involved in empathetic tasks, strengthening our hypothesis that alterations to its function and structure may be related to atypical social cognitive skills (Healy et al., 2013, Preston et al., 2007) .

Many studies of neural development have used maturation of face processing as a model of experience-expectant maturation, in which the developmental trajectory is tuned by features encountered in the environment during a sensitive developmental window (Fox et al., 2011, Leppänen and Nelson, 2009, Maurer and Werker, 2013). There are several aspects of the PT environment during early life that may pose challenges to this experience guided development. First, the coupling of face exposure and maturational state of the fusiform is asynchronous because environmental exposures occur much earlier in development for PT – and especially EPT – children than for FT youth. Second, EPT infants typically spend much of their early life in the Neonatal Intense Care Unit or other institutional settings, which are quite different from the environment of the typical newborn. This may impact development of the visual system (Fontana et al., 2020). Finally, for reasons which are not presently clear, EPT infants appear to adopt a different pattern of visual engagement with faces in early life (Berdasco-Muñoz et al., 2019, Santos et al., 2010, Telford et al., 2016). It is unclear whether this is causal or consequent to early wiring of the fusiform, however these differences in visual engagement may suggest that the development of face processing brain regions may begin to follow a different developmental trajectory relatively early in life.

The current study offers novel insight into the important interrelations between fusiform structure, function, and connectivity and the differences between EPT and FT youth. Our findings add to the growing body of literature investigating the neural correlates of altered social processing and social cognition and expand on the work limited work involving multiple modalities. While our comprehensive multimodal approach yields impressive results, our study is not without its limitations. First, despite being age and gender matched, our relatively small sample size may hinder generalizability to EPT youth outside of our sample. Indeed, previous studies have found conflicting findings regarding fusiform density among preterm (gestational age < 32 weeks) adolescents (Nosarti et al., 2008) and PT adults (Bäuml et al., 2015, Shang et al., 2019) , confirming the need for a large, representative sample. Relatedly, our convenience sample of FT youth recruited by flyers distributed to hospital staff may be a source of bias. It is unclear whether the group differences in the race of our samples reflects the different procedures used to recruit EPT and FT youth or if this reflects regional variation in race distributions of EPT and FT children. While estimated family income is an important factor in determining socioeconomic status, our lack of a complete measure detracts from our ability to accurately describe our samples. While future studies should include a representative sample from the community with no neurologic abnormalities, our sensitivity analyses suggest that the inclusion of these six participants did not hinder the current analyses. A second limitation is the cross-sectional design of our study. A longitudinal design is needed to further probe the developmental trajectory of the fusiform. The current study is unable to shed light on whether the density difference in the fusiform is the result of injury due to premature birth or an experience driven reduction as a sequalae of altered stimuli processing (Cassia et al., 2009). A third limitation is the use of neutral expression faces compared to a fixation cross. Without comparing to a non-face object, a possible interpretation of our findings is a difference in general visual perception instead of a specific alteration to face processing. Future studies should combine eye tracking with a fMRI face processing task to allow for direct comparison of neural activation and attention to faces. Further, the inclusion of five participants with different resting state scan acquisition parameters introduces a confound which future studies should avoid. Additionally, future work should use a multivariate approach to examine the relationship between brain structure, function, connectivity, and social outcomes. Despite these limitations, to our knowledge no other study has taken a multimodal approach to investigate fusiform differences in EPT and FT adolescents. A strength of this approach is to examine the cumulative impact of these features to form a more complete picture of how EPT youth process visual stimuli. Used in a clinical setting, this multimodal approach may help inform decisions regarding behavioral therapy, although replication on an independent sample is needed.

### 4.1 Conclusions

The current study used novel multimodal machine learning methods to determine with 95% accuracy whether an individual was born extremely premature or not – solely based on information from the right fusiform. While reporting partially overlapping regions of structural and functional differences is not unusual in this population, this study is, to our knowledge, the first to use multiple modalities including gray and white matter, BOLD signal during a face processing task, and functional connectivity both during the task and at rest to examine the effects of EPT birth on face processing. Our findings are further evidence of the long-term consequences of preterm birth and suggest that youth born EPT may have a differential mechanism for processing social stimuli than FT born youth.

#### CRediT authorship contribution statement

**Connor Grannis:** Conceptualization, Methodology, Software, Formal analysis, Investigation, Writing – original draft, Writing – review & editing, Visualization. **Andy Hung:** Methodology, Formal analysis, Writing – original draft, Investigation. **Roberto C. French:** Investigation, Resources. **Whitney I. Mattson:** Validation, Resources, Writing – review & editing, Supervision. **Xiaoxue Fu:** Writing – review & editing. **Kristen R. Hoskinson:** Writing – review & editing. **H. Gerry Taylor:** Writing – review & editing. **Eric E. Nelson:** Conceptualization, Writing – original draft, Writing – review & editing, Supervision, Project administration, Funding acquisition.

## Declaration of Competing Interest

The authors declare that they have no known competing financial interests or personal relationships that could have appeared to influence the work reported in this paper.
