# Supplementary material for: Multimodal classification of extremely preterm and term adolescents using the fusiform gyrus: A machine learning approach
Source: Neuroimage Clin. 2022 Jun 4;35:103078. doi: 10.1016/j.nicl.2022.103078 (PMC9189188; doi:10.1016/j.nicl.2022.103078)
Supplement: Supplementary data 1 [file mmc1.docx]

Supplemental Materials

S1


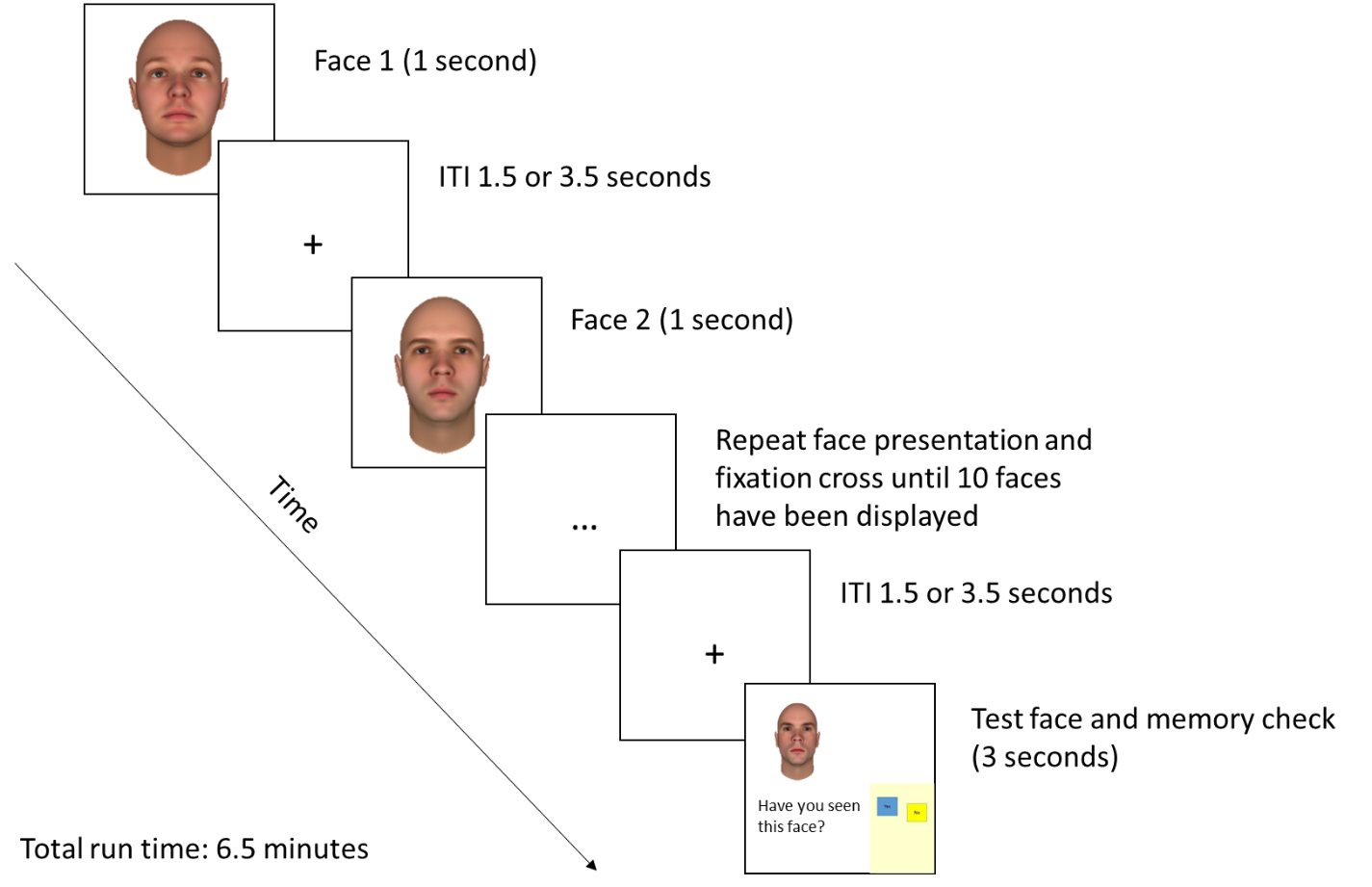


S1 displays the sequence of the face processing task.

S2

| Features included | Accuracy | Standard Deviation | *p*-value |
| --- | --- | --- | --- |
| BOLD, VBM, ReHo, RS, gPPI, PTX | 88.64 % | 31.74 | < 0.001 |
| BOLD, VBM, RS, gPPI, PTX | 86.36 % | 34.32 | < 0.001 |
| BOLD, VBM, ReHo, PTX | 84.09 % | 36.58 | < 0.001 |
| BOLD, VBM, ReHo, RS, PTX | 84.09 % | 36.58 | < 0.001 |
| BOLD, VBM, RS, PTX | 81.82 % | 38.57 | < 0.001 |
| BOLD, VBM, gPPI, PTX | 81.82 % | 38.57 | < 0.001 |
| BOLD, VBM | 79.55 % | 40.34 | < 0.001 |
| BOLD, VBM, ReHo | 79.55 % | 40.34 | < 0.001 |
| BOLD, VBM, RS | 79.55 % | 40.34 | < 0.001 |
| BOLD, VBM, gPPI | 79.55 % | 40.34 | < 0.001 |
| BOLD, VBM, PTX | 79.55 % | 40.34 | < 0.001 |
| BOLD, VBM, ReHo, RS | 79.55 % | 40.34 | < 0.001 |
| BOLD, VBM, ReHo, gPPI | 79.55 % | 40.34 | < 0.001 |
| BOLD, VBM, RS, gPPI | 79.55 % | 40.34 | < 0.001 |
| BOLD, VBM, ReHo, RS, gPPI | 79.55 % | 40.34 | < 0.001 |
| BOLD, VBM, ReHo, gPPI, PTX | 79.55 % | 40.34 | < 0.001 |
| BOLD | 77.27 % | 41.91 | < 0.001 |
| BOLD, ReHo | 75.00 % | 43.30 | < 0.001 |
| BOLD, ReHo, gPPI | 75.00 % | 43.30 | < 0.001 |
| BOLD, gPPI, PTX | 75.00 % | 43.30 | < 0.001 |
| BOLD, gPPI | 72.73 % | 44.54 | < 0.001 |
| BOLD, RS, gPPI | 72.73 % | 44.54 | 0.01 |
| BOLD, ReHo, gPPI, PTX | 72.73 % | 44.54 | 0.01 |
| BOLD, ReHo, RS, gPPI, PTX | 72.73 % | 44.54 | 0.01 |
| BOLD, RS | 70.45 % | 45.62 | 0.01 |
| BOLD, PTX | 70.45 % | 45.62 | 0.01 |
| BOLD, ReHo, RS, gPPI | 70.45 % | 45.62 | 0.02 |
| BOLD, RS, gPPI, PTX | 70.45 % | 45.62 | 0.02 |
| BOLD, ReHo, RS | 65.91 % | 47.40 | 0.04 |
| BOLD, ReHo, PTX | 63.64 % | 48.10 | 0.07 |
| BOLD, RS, PTX | 63.64 % | 48.10 | 0.07 |
| BOLD, ReHo, RS, PTX | 63.64 % | 48.10 | 0.08 |

S2 displays the accuracies, standard deviations, and *p*-values for each combination of features in the cluster identified in the BOLD analysis. Since this cluster was derived from the BOLD analysis, all combinations had to include BOLD.

S3

| Features included | Accuracy | Standard Deviation | *p*-value |
| --- | --- | --- | --- |
| BOLD, VBM, ReHo | 95.45 % | 20.83 | < 0.001 |
| BOLD, VBM, ReHo, PTX | 95.45 % | 20.83 | < 0.001 |
| VBM, ReHo | 93.18 % | 25.21 | < 0.001 |
| BOLD, VBM, gPPI | 93.18 % | 25.21 | < 0.001 |
| BOLD, VBM, PTX | 93.18 % | 25.21 | < 0.001 |
| BOLD, VBM, ReHo, gPPI | 93.18 % | 25.21 | < 0.001 |
| BOLD, VBM | 90.91 % | 28.75 | < 0.001 |
| VBM, gPPI | 90.91 % | 28.75 | < 0.001 |
| VBM, ReHo, RS | 90.91 % | 28.75 | < 0.001 |
| VBM, ReHo, gPPI | 90.91 % | 28.75 | < 0.001 |
| VBM, RS, gPPI | 90.91 % | 28.75 | < 0.001 |
| BOLD, VBM, ReHo, RS | 90.91 % | 28.75 | < 0.001 |
| BOLD, VBM, ReHo, gPPI, PTX | 90.91 % | 28.75 | < 0.001 |
| VBM | 88.64 % | 31.74 | < 0.001 |
| VBM, RS | 88.64 % | 31.74 | < 0.001 |
| BOLD, VBM, RS | 88.64 % | 31.74 | < 0.001 |
| VBM, gPPI, PTX | 88.64 % | 31.74 | < 0.001 |
| VBM, ReHo, RS, PTX | 88.64 % | 31.74 | < 0.001 |
| VBM, RS, gPPI, PTX | 88.64 % | 31.74 | < 0.001 |
| VBM, PTX | 86.36 % | 34.32 | < 0.001 |
| VBM, ReHo, PTX | 86.36 % | 34.32 | < 0.001 |
| BOLD, VBM, RS, gPPI | 86.36 % | 34.32 | < 0.001 |
| BOLD, VBM, RS, PTX | 86.36 % | 34.32 | < 0.001 |
| BOLD, VBM, gPPI, PTX | 86.36 % | 34.32 | < 0.001 |
| VBM, ReHo, RS, gPPI | 86.36 % | 34.32 | < 0.001 |
| VBM, ReHo, gPPI, PTX | 86.36 % | 34.32 | < 0.001 |
| BOLD, VBM, ReHo, RS, gPPI | 86.36 % | 34.32 | < 0.001 |
| BOLD, VBM, ReHo, RS, PTX | 86.36 % | 34.32 | < 0.001 |
| VBM, ReHo, RS, gPPI, PTX | 86.36 % | 34.32 | < 0.001 |
| BOLD, VBM, RS, gPPI, PTX | 84.09 % | 36.58 | < 0.001 |
| BOLD, VBM, ReHo, RS, gPPI, PTX | 84.09 % | 36.58 | < 0.001 |
| VBM, RS, PTX | 81.82 % | 38.57 | < 0.001 |

S3 displays the accuracies, standard deviations, and *p*-values for each combination of features in the cluster identified in the VBM analysis. Since this cluster was derived from the VBM analysis, all combinations include VBM.

S4 Validation of classification results within a leave-one-out framework

While intended to explore the primary findings, building classification models based on clusters in which group differences in an included feature are already observed likely biases the model towards inflated accuracy. To address this concern, the primary analyses were re-computed within a leave-one-participant-out framework. Secondary analyses and classifiers were subsequently computed and built based around the clusters that survived these analyses and model accuracy was evaluated on the left-out-participant. This process was repeated for each participant and accuracies were averaged across all participants. To assess the degree to which this occurred in our present implementation, we re-computed the *initial group difference* clusters for both BOLD and VBM using a leave-one-out framework. Using the same clustering criteria, we found comparable clusters of group differences both in BOLD response to faces and in gray matter density though the exact location was slightly different for each implementation (N=44). However, using this more conservative approach no clusters survived cluster thresholding for the gPPI analyses and clusters emerged in resting state in only 2/44 leave-one-out folds when the cluster from the VBM analysis was used as the seed. Instead of forcing this cluster to the rest of the folds, both gPPI and resting state were excluded from the subsequent classification using this implementation. This framework generates a single accuracy score per person (0=incorrect, 1=correct) per feature combination and does not easily support permutation testing. Therefore, only accuracy and standard deviation are listed in the tables below.

S4.1

| Features included | Accuracy | Standard Deviation |
| --- | --- | --- |
| BOLD, VBM | 77.27% | 42.39 |
| BOLD, VBM, ReHo | 77.27% | 42.39 |
| BOLD, VBM, PTX | 77.27% | 42.39 |
| BOLD, VBM, ReHo, PTX | 75.00% | 43.80 |
| BOLD | 70.45% | 46.15 |
| BOLD, PTX | 70.45% | 46.15 |
| BOLD, ReHo | 68.18% | 47.12 |
| BOLD, ReHo, PTX | 65.91% | 47.95 |

S4.1 displays the accuracies and standard deviations for each combination of features averaged across all participants in the clusters identified in the BOLD analyses within a leave-one-participant-out framework. Since this cluster was derived from the BOLD analyses, all combinations include BOLD.

S4.2

| Features included | Accuracy | Standard Deviation |
| --- | --- | --- |
| BOLD, VBM | 88.64% | 32.10 |
| BOLD, VBM, ReHo | 84.09% | 37.00 |
| BOLD, VBM, PTX | 84.09% | 37.00 |
| BOLD, VBM, ReHo, PTX | 84.09% | 37.00 |
| VBM | 79.55% | 40.80 |
| VBM, ReHo | 79.55% | 40.80 |
| VBM, ReHo, PTX | 79.55% | 40.80 |
| VBM, PTX | 72.73% | 45.05 |

S4.2 displays the accuracies and standard deviations for each combination of features averaged across all participants in the clusters identified in the VBM analyses within a leave-one-participant-out framework. Since this cluster was derived from the VBM analyses, all combinations include VBM.

S5

While the machine learning classifier was built to probe the two clusters from the primary analyses, there is a possibility that a multimodal classifier would be able to achieve such high accuracy in any brain region. To examine specificity of the right fusiform in discriminating EPT from FT youth, secondary and classification analyses were computed using the left supplementary motor area from the AAL atlas. This region was chosen as it is not a primary visual region. No clusters survived cluster thresholding for the gPPI or resting state analyses, which were excluded from the subsequent classification. Since this region was not identified through a primary analysis, all feature combinations are reported below.

S5

| Features included | Accuracy | Standard Deviation | *p*-value |
| --- | --- | --- | --- |
| BOLD, VBM, PTX | 65.91% | 47.40 | 0.05 |
| VBM | 61.36% | 48.69 | 0.08 |
| BOLD, VBM | 61.36% | 48.69 | 0.09 |
| BOLD, VBM, ReHo, PTX | 61.36% | 48.69 | 0.13 |
| VBM, ReHo | 59.09% | 49.17 | 0.14 |
| BOLD, PTX | 56.82% | 49.53 | 0.19 |
| ReHo | 54.55% | 49.79 | 0.52 |
| BOLD, VBM, ReHo | 54.55% | 49.79 | 0.32 |
| BOLD, ReHo, PTX | 54.55% | 49.79 | 0.32 |
| VBM, ReHo, PTX | 50.00% | 50.00 | 0.45 |
| VBM, PTX | 47.73% | 49.95 | 0.52 |
| PTX | 40.91% | 49.17 | 0.79 |
| ReHo, PTX | 38.64% | 48.69 | 0.80 |
| BOLD | 36.36% | 48.10 | 0.93 |
| BOLD, ReHo | 36.36% | 48.10 | 0.85 |

S5 displays the accuracies, standard deviations, and *p*-values for each combination of features in the left supplementary motor area
